# Resultados a longo prazo da angioplastia de artérias renais com stent na doença aterosclerótica: revisão sistemática

**DOI:** 10.1590/1677-5449.010816

**Published:** 2017

**Authors:** Daniel Emilio Dalledone Siqueira, Ana Terezinha Guillaumon

**Affiliations:** 1 Universidade Estadual de Campinas – UNICAMP, Cirurgia Vascular, Campinas, SP, Brasil.

**Keywords:** angioplastia, aterosclerose, hipertensão renovascular, nefropatia, obstrução da artéria renal

## Abstract

A doença renovascular aterosclerótica é a principal causa de hipertensão secundária. A história natural da doença demonstra taxas de progressão de 4 a 12% ao ano. Entre os métodos de tratamento existe a angioplastia com stent de artérias renais; porém, poucos estudos clínicos demonstraram seus resultados a longo prazo. Esta revisão sistemática da literatura se propõe a apresentar os resultados a longo prazo (acima de 24 meses) da angioplastia com stent de artérias renais na doença aterosclerótica em relação à função renal e aos níveis pressóricos no controle da hipertensão. Foi realizada uma ampla pesquisa, utilizando os termos apropriados, nas bases de dados LILACS, EMBASE, SCIELO, Cochrane Library e MEDLINE. De um total de 2.170 referências, apenas sete artigos contemplavam todos os critérios de inclusão. Conclui-se que, a longo prazo, há uma estabilização da função renal, redução dos níveis pressóricos e diminuição do número de classes de medicamentos anti-hipertensivos.

## INTRODUÇÃO

Estenose das artérias renais (EAR) pode ser definida como uma doença multifatorial, de diversas etiologias, que acomete a vasculatura arterial renal de maneira unilateral ou bilateral, determinando graus de estenose, desde a sua origem até sua porção hilar. Pode apresentar-se clinicamente como hipertensão renovascular e doença isquêmica renal, levando a complicações sistêmicas a longo prazo^1^^,^ 2. Essa patologia está associada a um maior risco cardiovascular e à elevação da mortalidade^3^^-^ 6. Para fins de pesquisa e tratamento, diversos autores consideram a estenose crítica da artéria renal como aquela maior que 60%^7^. Porém, existem muitas divergências na literatura, com autores considerando estenose crítica toda estenose maior que 50%^1^. Através dos métodos diagnósticos e laboratoriais, bem como do avanço tecnológico dos exames não invasivos, nos últimos anos há um aumento dos casos diagnosticados, permitindo medidas terapêuticas mais precoces.

Existem muitas controvérsias relativas ao tratamento da doença aterosclerótica renal, sem haver consenso na literatura^8^^-^ 10. Existem estudos divergentes relativos ao tratamento clínico *versus* angioplastia, sendo a grande maioria com tempo de seguimento curto. Poucos estudos na literatura apresentam e discorrem sobre os resultados clínicos tardios (acima de 24 meses) da angioplastia com stent de artéria renal.

A prevalência da EAR é controversa, pois faltam estudos populacionais voltados à identificação e correlação com raça, idade e sexo. Porém, estudos demonstraram que, em algumas populações, a EAR está presente em 1 a 5% das pessoas com hipertensão arterial sistêmica^1^ e representa a principal causa de hipertensão secundária. Além disso, estima-se que seja responsável por 1% dos casos de hipertensão leve a moderada e por 10 a 40% dos casos de hipertensão aguda, severa ou refratária^11^. Pesquisas populacionais sugerem que a prevalência em pessoas acima de 65 anos de idade é maior que 7%^1^.

A EAR pode ter diversas etiologias, porém as duas principais causas são a aterosclerose e a displasia fibromuscular. A origem aterosclerótica é a mais frequente, representando 70 a 80% dos casos, acomete mais homens acima de 40 anos de idade e gera estenoses nos segmentos proximais das artérias renais^12^.

A história natural da doença aterosclerótica renal não está completamente elucidada, porém se sabe que há uma estenose progressiva, com redução do fluxo arterial e consequente perda da função renal^13^^,^ 14. Essa perda é diretamente dependente do grau de estenose da artéria renal. Estima-se que a doença aterosclerótica renovascular cause, a cada ano, falência renal em 5 a 15% dos adultos que se tornam dialíticos^15^, sendo que apenas 56% desses pacientes que se tornaram dialíticos permanecem vivos por mais de 2 anos^15^.

Diante do exposto, e tendo-se em vista que a doença aterosclerótica renovascular é a principal causa de hipertensão arterial sistêmica secundária e que o manejo terapêutico por meio de intervenção cirúrgica apresenta uma série de controvérsias, demonstra-se haver uma carência de estudos clínicos e desfechos esclarecedores. Além disso, percebe-se a existência de pesquisas com resultados diversos e conflitantes, levando à falta de dados conclusivos.

Nesse âmbito, faz-se necessário um estudo de revisão sistemática a fim de esclarecer o desfecho a longo prazo da angioplastia de artérias renais no tratamento da doença renovascular aterosclerótica. A revisão sistemática é uma síntese dos mais diversos estudos acerca de uma questão específica. Sua realização é clara, com metodologia predeterminada e reprodutível. Difere em muito das revisões narrativas, conhecidas como revisões clássicas, pois segue os preceitos de um ensaio científico convencional.

O objetivo deste estudo foi avaliar e resumir criteriosamente, a partir de uma revisão sistemática da literatura, o desfecho clínico a longo prazo (acima de 24 meses), referente à função renal e aos níveis pressóricos, das angioplastias com utilização de stent nas artérias renais para o tratamento da doença renovascular de origem aterosclerótica.

## MÉTODOS

Revisão sistemática da literatura para combinar e analisar os dados. Foram utilizados os métodos recomendados pela Colaboração Cochrane.

### Considerações éticas

Não foram utilizados dados confidenciais ou pessoais nem foram realizadas pesquisas em seres humanos pelos pesquisadores. Toda pesquisa foi baseada exclusivamente em estudos clínicos primários publicados em bases de dados eletrônicas. Vale lembrar que a Comissão Nacional de Ética em Pesquisa (CONEP) tem dado pareceres referentes a revisões sistemáticas no sentido de que as pesquisas envolvendo apenas dados de domínio público, que não identifiquem os participantes da pesquisa ou que sejam apenas revisão bibliográfica e sem envolvimento de seres humanos não necessitam de aprovação por parte do sistema CEP-CONEP.

### Local e período da pesquisa

Esta pesquisa foi desenvolvida e realizada na Disciplina de Moléstias Vasculares do Hospital de Clínicas da UNICAMP. A busca por referências foi realizada do período de abril a agosto de 2016.

Critérios de inclusão dos artigos:

- Tipos de estudos incluídos:

Foram incluídos na presente pesquisa os estudos clínicos prospectivos ou retrospectivos que avaliaram os resultados do tratamento endovascular da estenose de artérias renais de origem aterosclerótica através de angioplastia primária com stent, com seguimento médio de no mínimo 24 meses.

- Tipos de estudos excluídos:

Foram excluídos da presente pesquisa todos os estudos clínicos que não preenchiam os critérios de inclusão, tais como aqueles com seguimento médio menor que 24 meses; outras etiologias da estenose renal que não sejam aterosclerose; tratamento com angioplastia com balão, angioplastia sem implante de stent, angioplastia com uso de mecanismo mecânico de proteção renal (filtro de embolização); casos de re-estenose submetidos a patência primária assistida ou patência secundária; estudos clínicos comparativos entre angioplastia com implante de stent. Além disso, foram excluídos relatos de caso e tratamento medicamentoso em que os dados do grupo angioplastia com stent não foram descritos separadamente ou de maneira clara.

### Pacientes

Pacientes com estenose de artéria renal de origem aterosclerótica tratados por via endovascular com colocação de stent.

### Tipos de intervenção

Foram selecionados estudos apenas com intervenção endovascular no tratamento da estenose de artérias renais. Foram excluídas pesquisas clínicas com outros tipos de intervenção.

### Desfechos clínicos avaliados nos estudos

Foram avaliados os seguintes desfechos clínicos: função renal, níveis pressóricos, número de classes de anti-hipertensivos, taxa de re-estenose, número de pacientes dialíticos.

Estratégias de busca para seleção dos artigos:

- Bases de dados eletrônicas:

Estudos relevantes foram pesquisados e identificados nas principais bases de dados informatizadas na área da saúde, entre elas: Literatura Latina Americana e do Caribe em Ciências da Saúde (LILACS), Excerpta Medica Database (EMBASE), Cochrane Library, Scientific Eletronic Library Online (SciELO) e MEDLINE por meio da PubMed.

Buscas eletrônicas:

A busca nas bases de dados eletrônicas foi realizada com os termos em inglês descritos a seguir: *renal artery, stenosis, angioplasty, atherosclerotic, stent angioplasty, stent balloon, atherosclerotic, long term*.

Seleção dos estudos:

No desenvolvimento desta pesquisa, os dois autores responsáveis pela revisão da literatura (DEDS e ATG) avaliaram de forma independente os títulos e resumos identificados pela pesquisa eletrônica. Foram obtidas cópias do texto completo de todos os ensaios clínicos potencialmente relevantes e/ou relevantes, os quais foram avaliados de acordo com os critérios de inclusão dos artigos.

Depois que todas as cópias completas dos textos dos artigos potencialmente ou definitivamente relevantes foram obtidas, os dois autores analisaram e classificaram os estudos da seguinte forma: estudos excluídos, estudos para análise, estudos incluídos.

Não houve mascaramento dos autores dos artigos, instituições e dos resultados dos ensaios durante a avaliação.

Forma de lidar com a falta de dados:

Considerando-se a falta de dados para a pesquisa nos estudos selecionados, buscou-se um contato com o investigador principal dos estudos clínicos. Essa medida foi realizada em função da falta de estatísticas ou dados nos estudos selecionados. Porém, tornou-se inviável a obtenção de informações adicionais. Diante disso, todos os dados foram extraídos a partir dos dados presentes nos documentos publicados.

Busca por estudos em andamento:

Na Biblioteca Cochrane verificou-se que não havia nenhuma revisão sistemática em andamento com o mesmo tema proposto pelo presente trabalho.

Buscas de estudos não publicados:

Não foi realizada busca de estudos não publicados, tendo-se em vista que a presente revisão sistemática incluiu estudos clínicos e dados já publicados.

Avaliação crítica dos estudos:

A avaliação crítica da qualidade dos estudos baseou-se nos seguintes aspectos: definição clara dos objetivos iniciais e dos desfechos clínicos medidos, avaliação da qualidade metodológica do estudo, utilização de métodos estatísticos apropriados, descrição se houve cálculo do tamanho da amostra a ser estudada, descrição se o estudo foi uni-institucional ou multicêntrico, descrição das fontes de financiamento dos estudos.

Extração dos dados:

Após seleção dos artigos elegíveis para o trabalho, foi realizada uma leitura atenta e cuidadosa, com a finalidade de extrair os dados relevantes. Essa extração levou sempre em consideração as características dos estudos e a compilação dos resultados de cada artigo, utilizando-se de um protocolo de coleta de dados.

Foram confeccionadas planilhas, as quais foram submetidas a pré-teste com cinco estudos da mesma área, mas não referentes a essa revisão. Durante esse pré-teste não foi detectada nenhuma ambiguidade ou falha, sendo as planilhas aprovadas para uso na pesquisa principal.

Para identificação de cada trabalho dentro da revisão, foi utilizado o nome do primeiro autor de cada artigo. Dos estudos, foram coletados os dados que permitiram os cálculos dos testes estatísticos propostos e outros dados julgados como relevantes.

Todos os dados utilizados na presente revisão foram coletados diretamente dos artigos publicados ou calculados através das informações disponíveis.

Análise e interpretação dos dados:

Devido às peculiares características dos estudos clínicos no que refere ao tamanho da amostra, dados heterogêneos, além de dados clínicos ausentes, foram construídos gráficos e tabelas de maneira a permitir a comparação dos dados clínicos avaliados, sempre com o propósito de descrever as informações tratadas de maneira clara e eficiente.

Os dados extraídos dos estudos clínicos incluídos na presente revisão sistemática foram descritos quanto a números absolutos e frequências. Por não se tratar de um estudo para o qual se fazem inferências, uma vez que não se trata de amostra única, os resultados obtidos apresentam todo o material disponível na literatura para a metodologia adotada nesta pesquisa.

## RESULTADOS

Inicialmente foram encontrados na literatura 2.170 referências, sendo 324 registros duplicados, totalizando efetivamente 1.684 referencias únicas nas bases de dados eletrônicas pesquisadas. Após avaliação do título, foram excluídos 1.337 artigos, sendo selecionados 347 estudos clínicos de potencial interesse. Foi realizada a leitura do resumo desses 347 artigos, com identificação de 88 estudos com potencial de preencher os critérios de seleção dos estudos. Após leitura e avaliação inicial, foram excluídos 62 artigos e selecionados 26 artigos.

Todos os 26 artigos foram rigorosamente avaliados conforme protocolo de inclusão da pesquisa, com análise criteriosa. Dos 26 artigos previamente identificados, foram excluídos 19 artigos e selecionados sete estudos que preenchiam efetivamente todos os critérios de inclusão da presente pesquisa.

**Figura 1:**
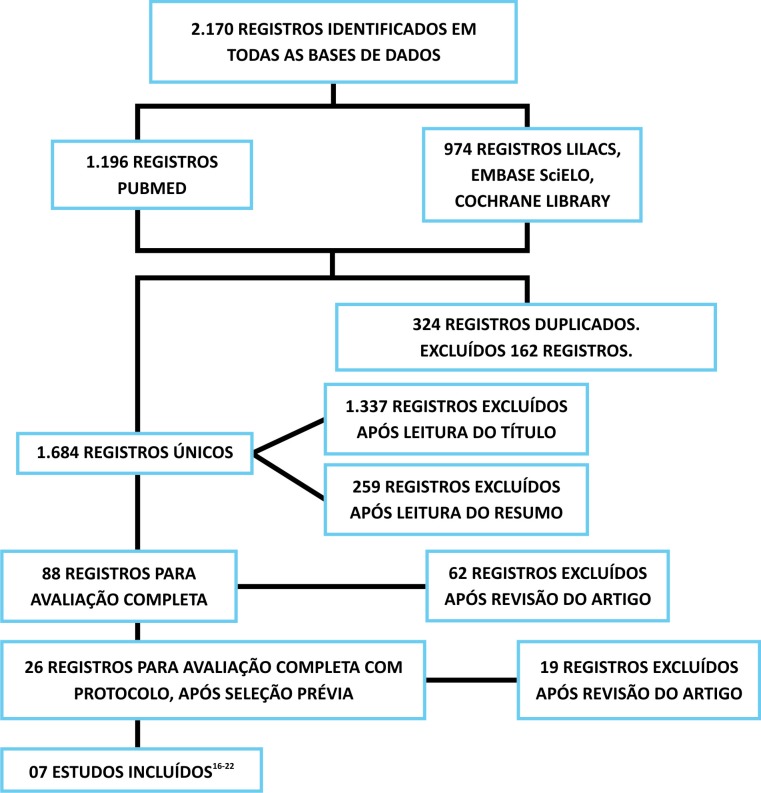
Resumo dos resultados das buscas nas bases eletrônicas.

A representação esquemática do resultado da busca de estudos encontra-se na Figura 1, a qual demonstra os números de artigos identificados, selecionados, excluídos e incluídos nesta revisão sistemática.

Qualidade metodológica dos estudos incluídos:

Entre os estudos selecionados e incluídos na presente revisão sistemática após análise dos critérios de inclusão, todos são retrospectivos e com único grupo intervenção. Diante disso, não houve mascaramento dos avaliadores, bem como cálculo do tamanho amostral, manejo das perdas de seguimento, critérios de alocação e análise por intenção de tratamento por parte dos autores das pesquisas.

Análise dos dados:

- Características das amostras dos estudos:

Após análise dos estudos, conforme metodologia descrita, foram incluídos sete estudos clínicos^16^^-^ 22, totalizando 705 pacientes. As amostras incluíram 375 pacientes do sexo masculino e 249 do sexo feminino, porém Zhao et al.^19^ não estratificaram o sexo dos participantes em seu estudo com 81 pacientes (Tabela 1). A idade média dos pacientes foi de 68,8 anos, variando de 37 a 87 anos (Tabela 2).

**Tabela 1:** Características das amostras dos estudos clínicos incluídos na revisão sistemática, demonstrando o n de cada estudo e a distribuição quanto ao sexo.

**Artigo**	**n**	**Masculino**		**Feminino**	
		**n**	**%**	**n**	**%**
Rodriguez Lopez et al.^16^	108	64	59,3	44	40,7
Fiala et al.^17^	21	11	52,4	10	47,6
Ruchin et al.^18^	89	53	59,6	36	40,4
Zhao et al.^19^	81	ND		ND	
Chuan-jun et al.^20^	125	74	59,2	51	40,8
Zeller et al.^21^	241	153	63,5	88	36,5
Bucek et al.^22^	40	20	50,0	20	50,0

ND: Dados não disponíveis no estudo primário.

**Tabela 2:** Características das amostras dos estudos clínicos incluídos na revisão sistemática, demonstrando o n de cada estudo e a distribuição quanto à idade média, idade mínima e idade máxima em anos.

**Artigo**	**n**	**Idade média**	**Idade mínima**	**Idade máxima**
Rodriguez Lopez et al.^16^	108	72,0	37,0	87,0
Fiala et al.^17^	21	63,0	46,0	87,0
Ruchin et al.^18^	89	70,0	37,0	86,0
Zhao et al.^19^	81	76,2	ND	ND
Chuan-jun et al.^20^	125	66,4	ND	ND
Zeller et al.^21^	241	67,0	44,0	84,0
Bucek et al.^22^	40	65,2	ND	ND

ND: Dados não disponíveis no estudo primário.

Características dos estudos

Quanto ao país de origem dos estudos temos: dois artigos dos Estados Unidos da América, dois da China, um da Austrália, um da Alemanha e um da Áustria. O nome das revistas em que os artigos selecionados foram publicados é demonstrado no Quadro 1.

**Quadro 1:** Nome dos autores, ano de publicação do artigo, país de origem, centros e nome da revista.

**Autor principal**	**Ano**	**País de origem**	**Centros**	**Nome da revista/publicação**	**Seguimento (meses)**
Rodriguez Lopez et al.^16^	1999	Estados Unidos	Unicêntrico	Journal of Vascular Surgery	36
Fiala et al.^17^	1998	Estados Unidos	Multicêntrico	Annals of Vascular Surgery	24
Ruchin et al.^18^	2007	Austrália	Unicêntrico	Heart, Lung and Circulation	28
Zhao et al.^19^	2012	China	Unicêntrico	Clinical Interventions in Aging	31,3
Chuan-jun et al.^20^	2012	China	Unicêntrico	Chinese Medical Journal	24
Zeller et al.^21^	2003	Alemanha	Unicêntrico	Journal of Endovascular Therapy	27
Bucek et al.^22^	2003	Áustria	Unicêntrico	Wiener Klinische Wochenschrift	39,6

Entre os estudos clínicos incluídos na presente revisão sistemática, os sete artigos são retrospectivos, com seis estudos realizados em um único centro e um estudo multicêntrico. O Quadro 1 apresenta o tipo de centro onde foram realizados os estudos publicados conforme autor e ano^16^^-^ 22. Além disso, o tempo de seguimento médio dos estudos foi de 29,9 meses, variando de 24 a 39,6 meses (Quadro 1). A seleção, os procedimentos de angioplastia com stent e acompanhamento clínico nesses estudos foram realizados dos anos de 1993 a 2010, apesar de as publicações terem sido realizadas dos anos de 1998 a 2012.

Artérias tratadas/Tipos de stents/lateralidade:

Dos 705 pacientes, foram tratadas 876 artérias, com 901 stents renais implantados, representando nos estudos uma média de 1,0 a 1,57 stents por paciente e 1,0 a 1,32 stents por artéria (Tabela 3). Quanto aos stents utilizados nos estudos clínicos, apenas os autores Rodriguez Lopez^16^, Fiala et al.^17^ e Zeller et al.^21^ fazem referência à marca e ao modelo do stent utilizado. Os demais artigos apenas descrevem a utilização de stent balão expansível na totalidade dos procedimentos realizados.

**Tabela 3:** Número total de pacientes por estudo clínico, número de artérias renais tratadas, número de stents, média de stents por artéria e por paciente nos respectivos estudos clínicos.

**Artigo**	**n**	**Número de artérias tratadas**	**Número de stents implantados**	**Número de stents por artéria**	**Número de stents por paciente**
Rodriguez Lopez et al.^16^	108	125	125	1,15	1,15
Fiala et al.^17^	21	25	33	1,32	1,57
Ruchin et al.^18^	89	102	110	1,08	1,25
Zhao et al.^19^	81	86	86	1,00	1,06
Chuan-jun et al.^20^	125	143	143	1,00	1,14
Zeller et al.^21^	241	355	364	1,02	1,51
Bucek et al.^22^	40	40	40	1,00	1,00

Entre as amostras dos estudos incluídos nesta revisão, observou-se um predomínio de casos de estenose de artérias renais unilateral, representando 76,5% do total de pacientes. Os artigos não fazem referência a casos de rim único presentes nas amostras. Além disso, os estudos apresentam discordância no grau de estenose mínimo considerado para tratamento endovascular com implante de stent dos pacientes, variando de 60 a 70%.

Indicações dos procedimentos:

Quanto às indicações clínicas para a realização do procedimento endovascular, observa-se uma distribuição entre pacientes hipertensos sem nefropatia isquêmica, hipertensão associada a nefropatia isquêmica e insuficiência renal dialítica (Tabela 4). De todos os estudos, apenas um paciente apresentava insuficiência renal dialítica, incluído no estudo de Fiala et al.^17^, o qual não apresentou nenhuma melhora após o procedimento, tanto em termos de função renal quanto em mudança do controle pressórico. Com relação aos procedimentos endovasculares realizados, apenas Rodriguez Lopez^16^ e Zeller et al.^21^ discorrem sobre as vias de acesso arteriais para realização do procedimento, havendo predomínio do acesso femoral, seguido pelo acesso braquial.

**Tabela 4:** Indicações clínicas para a realização do procedimento endovascular.

**Artigo**	**n**	**Hipertensos**		**Hipertensão renovascular isolada (sem nefropatia isquêmica)**		**Nefropatia isquêmica (insuficiência renal não dialítica) + hipertensão**		**Insuficiência renal dialítica**	
		**n**	**%**	**n**	**%**	**n**	**%**	**n**	**%**
Rodriguez Lopez et al.^16^	108	96	88,9	64	59,3	32	29,6	0	0,0
Fiala et al.^17^	21	18	85,7	11	52,4	8	38,1	1	4,8
Ruchin et al.^18^	89	77	86,5	21	23,6	56	62,9	0	0,0
Zhao et al.^19^	81	81	100,0	10	12,3	71	87,7	0	0,0
Chuan-jun et al.^20^	125	118	94,4	79	63,2	39	31,2	0	0,0
Zeller et al.^21^	241	239	99,2	ND	ND	33	13,7	0	0,0
Bucek et al.^22^	40	40	100,0	ND	ND	10	25,0	0	0,0

ND: Dados não disponíveis no estudo primário.

- Função renal

Diante da função renal no pós-procedimento, alguns autores descreveram os desfechos clínicos que puderam ser estratificados em relação à função renal basal em piora, estabilização e melhora da função renal, sendo que todos esses dados referem-se ao término do seguimento clínico do estudo (Tabela 5). Os níveis de creatinina pré-procedimento, pós procedimento em até 30 dias e tardio são demonstrados em mg/dL (Tabela 6).

**Tabela 5:** Desfecho clínico de cada estudo no que refere-se à função renal.

**Artigo**	**n**	**Piora**		**Estabilização**		**Melhora**	
		**n**	**%**	**n**	**%**	**n**	**%**
Rodriguez Lopez et al.^16^	108	5	4,6	103	95,4	0	0,0
Fiala et al.^17^	21	1	4,8	13	61,9	7	33,3
Ruchin et al.^18^	89	ND	ND	ND	ND	ND	ND
Zhao et al.^19^	81	13	16,0	26	32,1	8	9,9
Chuan-jun et al.^20^	125	23	18,4	56	44,8	31	24,8
Zeller et al.^21^	241	ND	ND	ND	ND	ND	ND
Bucek et al.^22^	40	10	25,0	ND	ND	ND	ND

ND: Dados não disponíveis no estudo primário.

**Tabela 6:** Níveis de creatinina em mg/dL pré-procedimento, pós-procedimento (até 30 dias) e tardio.

**Artigo**	**Pré-procedimento**		**Pós-procedimento (até 30 dias)**		**p**	**Tardio**		**p**
	**n**	**%**	**n**	**%**		**n**	**%**	
Rodriguez Lopez et al.^16^	2		1,8		ND	ND		ND
Fiala et al.^17^	1,47	0,57	ND		ND	1,31	0,41	0,076
Ruchin et al.^18^	1,58	0,07	ND		ND	1,47	0,68	0,16
Zhao et al.^19^	1,46	0,63	ND		ND	ND		ND
Chuan-jun et al.^20^	1,66	1,04	1,71	1,08	>0,05	1,77	1,15	> 0,05
Zeller et al.^21^	ND		ND		ND	ND		ND
Bucek et al.^22^	ND		ND		ND	1,3	0,4	ND

ND: Dados não disponíveis no estudo primário.

- Níveis pressóricos

Quanto à pressão arterial sistêmica, foram avaliados o número de classes anti-hipertensivas utilizadas nos pacientes no pré e pós-operatório tardio (Tabela 7), níveis de pressão arterial média (PAM), pressão arterial sistólica (PAS) e pressão arterial diastólica (PAD) (Tabelas 8, 9 e 10).

**Tabela 7:** Número de classes anti-hipertensivas no pré e pós-operatório tardio.

**Artigo**	**Pré-procedimento**		**Pós-procedimento (tardio)**		**p**
	**n**	**%**	**n**	**%**	
Rodriguez Lopez et al.^16^	ND		ND		ND
Fiala et al.^17^	3,1	0,13	2,7	0,25	0,056
Ruchin et al.^18^	3,14	1,65	2,62	1,39	0,05
Zhao et al.^19^	2,28	1,18	2,1	1,0	< 0,01
Chuan-jun et al.^20^	2,7	1,1	1,6	1,1	< 0,05
Zeller et al.^21^	ND		ND		ND
Bucek et al.^22^	ND		ND		ND

ND: Dados não disponíveis no estudo primário.

**Tabela 8:** Níveis de pressão arterial média em mmHg.

**Artigo**	**Pré-procedimento**		**Pós-procedimento (até 30 dias)**		**p**	**Tardio**		**p**
	**n**	**%**	**n**	**%**		**n**	**%**	
Rodriguez Lopez et al.^16^	ND		ND		ND	ND		ND
Fiala et al.^17^	117	13,4	ND		ND	113	12,8	0,002
Ruchin et al.^18^	ND		ND		ND	ND		ND
Zhao et al.^19^	ND		ND		ND	ND		ND
Chuan-jun et al.^20^	ND		ND		ND	ND		ND
Zeller et al.^21^	ND		ND		ND	ND		ND
Bucek et al.^22^	ND		ND		ND	ND		ND

ND: Dados não disponíveis no estudo primário.

**Tabela 9:** Níveis de pressão arterial sistólica em mmHg.

**Artigo**	**Pré-procedimento**		**Pós-procedimento (até 30 dias)**		**p**	**Tardio**		**p**
	**n**	**%**	**n**	**%**		**n**	**%**	
Rodriguez Lopez et al.^16^	ND		ND		ND	ND		ND
Fiala et al.^17^	ND		ND		ND	ND		ND
Ruchin et al.^18^	161,7	29,5	138,2	20,5	< 0,0001	138,7	7,9	< 0,0001
Zhao et al.^19^	155,9	22,8	130,3	4,5	< 0,01	135	4,7	< 0,01
Chuan-jun et al.^20^	168	23	138	17	< 0,05	141	20	< 0,05
Zeller et al.^21^	ND		ND		ND	ND		ND
Bucek et al.^22^	ND		ND		ND	ND		ND

ND: Dados não disponíveis no estudo primário.

**Tabela 10:** Níveis de pressão arterial diastólica em mmHg.

**Artigo**	**Pré-procedimento**		**Pós-procedimento (até 30 dias)**		**p**	**Tardio**		**p**
	**n**	**%**	**n**	**%**		**n**	**%**	
Rodriguez Lopez et al.^16^	ND		ND		ND	ND		ND
Fiala et al.^17^	ND		ND		ND	ND		ND
Ruchin et al.^18^	78,4	13,8	69,0	11,6	< 0,003	76,7	10,8	0,62
Zhao et al.^19^	79,3	10,8	66,7	8,9	< 0,01	68,3	10,2	< 0,01
Chuan-jun et al.^20^	92	12	78	10	< 0,05	80	11	< 0,05
Zeller et al.^21^	ND		ND		ND	ND		ND
Bucek et al.^22^	ND		ND		ND	ND		ND

ND: Dados não disponíveis no estudo primário.

- Perda de seguimento

Dos estudos observou-se uma perda da amostra no seguimento (Tabela 11).

**Tabela 11:** Número inicial de pacientes, número de pacientes no follow-up e taxa de perda dos pacientes.

**Artigo**	**n**	**Tempo de seguimento (meses)**	**Número de pacientes inicial**	**Número de pacientes no seguimento**	**Perda (%)**
Rodriguez Lopez et al.^16^	108	36	108	89	17,6
Fiala et al.^17^	21	24	21	16	23,8
Ruchin et al.^18^	89	28	89	81	9,0
Zhao et al.^19^	81	31,3	81	47	42,0
Chuan-jun et al.^20^	125	24	125	110	12,0
Zeller et al.^21^	241	27	241	198	17,8
Bucek et al.^22^	40	39,6	40	40	0,0

## DISCUSSÃO

Esta revisão sistemática da literatura representa a evidência científica mais atualizada sobre os resultados da angioplastia com stent na doença aterosclerótica das artérias renais a longo prazo, com período mínimo de seguimento de 24 meses. Até o presente momento, nenhum estudo se propôs a reunir por meio de uma revisão sistemática ou metanálise os resultados tardios da angioplastia.

Algumas considerações sobre a qualidade metodológica dos estudos incluídos nesta revisão devem ser feitas. Dos estudos disponíveis na literatura, há uma heterogeneidade de metodologias utilizadas e desfechos clínicos avaliados. Tendo-se em vista os estudos primários que preencheram os critérios de inclusão, todos são retrospectivos. Além disso, dentre os ensaios clínicos prospectivos, o tempo de seguimento é curto, ou seja, menor que 24 meses^1^^,^ 12.

Em 1836, no Guy’s Hospital, em Londres, Richard Bright descreveu a presença de hipertensão arterial e doença parenquimatosa renal. Ele observou, em autópsias, que pacientes com alterações no parênquima renal tinham um aumento das camâras cardíacas^23^. Essa observação foi o estímulo inicial para que, em 1871, Traube descrevesse de maneira especulativa um processo mecânico em que o coração, devido ao aumento pressórico, deveria fazer mais força contrátil para o sangue passar pelas porções distais do sistema vascular, levando a uma hipertrofia miocárdica^24^. A descrição inicial de Bright levou à tentativa de vários autores de recriarem os achados clínicos em modelos experimentais. Foi Harry Goldblatt, em 1934, que demonstrou que a constrição das artérias renais era o evento inicial, desencadeando hipertensão arterial, atrofia renal e hipertrofia cardíaca^25^.

O primeiro tratamento descrito foi a nefrectomia para o tratamento da hipertensão renovascular, tendo-se em vista a inexistência de técnicas arteriais reconstrutivas. Tratava-se de um método definitivo e com alta morbimortalidade. Com a evolução das técnicas, ao longo do século XX desenvolveram-se os métodos de revascularização renal através de endarterectomia e posteriormente enxerto vascular. Essas modalidades permaneceram vigentes e soberanas até o final da década de 70. Por meio dos inventos de Grüntzig em 1978, que descreveu a angioplastia com balão, possibilitando o tratamento endovascular, houve uma revolução nos métodos terapêuticos^26^.

Na década de 80 predominaram estudos clínicos no intuito de demonstrar, nas mais diversas etiologias da doença estenótica das artérias renais, os resultados das duas modalidades de tratamento: cirurgia aberta *versus* angioplastia com balão. Diversos estudos demonstraram a superioridade dos procedimentos endovasculares, com menores índices de morbidade^1^.

Com o passar dos anos, na década de 90, os resultados da angioplastia com balão se mostraram insatisfatórios, com altos índices de re-estenose e necessidade de novas intervenções endovasculares na doença aterosclerótica^27^. Iniciaram-se nessa época os estudos com uma nova modalidade de tratamento, a angioplastia com stent, que passou a representar a principal opção de técnica na doença aterosclerótica, com resultados superiores aos da cirurgia aberta e da angioplastia com balão^28^.

Há pouco mais de uma década, com o avanço da indústria farmacêutica e o desenvolvimento de novas moléculas e fármacos, houve o início de um movimento mundial no intuito de comparar os benefícios do tratamento clínico ao tratamento intervencionista. Isso determinou o início de ensaios clínicos prospectivos em diversos países. Porém, muitos estudos apresentam falhas metodológicas graves, colocando sob questionamento os resultados demonstrados.

Entre os grandes estudos clínicos, podemos citar o *Stent placement in patients with atherosclerotic renal artery stenosis and impaired renal function: a randomized trial* (estudo STAR), no qual foi realizado implante de stent apenas em uma parte do grupo intervenção e foi realizada angioplastia como medida de resgate no grupo tratamento clínico em casos de hipertensão arterial resistente, maligna e edema agudo de pulmão^29^. Já no estudo *Angioplasty and Stenting for Renal Artery Lesions* (ASTRAL), houve uma variabilidade no grau de estenose para indicação do procedimento e pacientes foram excluídos no início do estudo caso houvesse necessidade de revascularização^30^. No estudo *Cardiovascular Outcomes in Renal Atherosclerotic Lesions* (CORAL), houve uma otimização excessiva das medidas clínicas, foi utilizado filtro de proteção de microembolização, e foram excluídos pacientes que foram hospitalizados por insuficiência cardíaca nos últimos 30 dias prévios à admissão do estudo^1^. Sabe-se que o edema agudo de pulmão e a insuficiência cardíaca são agravados com a estenose de artérias renais^1^^,^ 11.

Até o presente momento não existem diretrizes sobre a indicação do tratamento clínico ou cirúrgico da doença renovascular^8^^,^ 10. Existem apenas diretrizes da American Heart Association e da American College of Cardiology para indicações de rastreamento populacional.

Mas o maior problema de todas as pesquisas até o momento publicadas concerne no fato de não discorrerem sobre os resultados tardios, tendo-se em vista que o período de seguimento médio é curto e portanto não reflete os resultados a longo prazo. Isso porque, a partir do momento da indicação do implante de um stent, um dos principais questionamentos será o desfecho tardio.

Diversos serviços têm baseado suas condutas exclusivamente em ensaios clínicos como os já mencionados STAR^29^, ASTRAL^30^ e CORAL^1^, sem terem uma visão crítica desses ensaios, o que pode gerar condutas e resultados conflitantes a longo prazo, tendo-se em vista que a doença aterosclerótica renal, quando acomete de maneira bilateral as artérias, associa-se a taxa de mortalidade de 52% em 4 anos^31^^,^ 32. Isso demonstra que há necessidade da realização de alguma medida clínica imediata no momento do diagnóstico, no intuito de modificar a história natural da doença.

Dos estudos clínicos incluídos na presente revisão sistemática, apenas o estudo de Fiala et al.^17^ era multicêntrico, e todos eram retrospectivos. Estes últimos apresentam um nível de evidência mais baixo em relação aos estudos prospectivos, porém permitem que sejam discutidos os métodos empregados, os procedimentos instaurados e os resultados apresentados.

Dos pacientes tratados nos estudos selecionados na presente revisão sistemática, há uma predominância de pacientes do sexo masculino na sexta década de vida^33^. Esse fato coincide com o pico de incidência da doença aterosclerótica, a exemplo de outros territórios arteriais, como coronárias e carótidas. Porém, observam-se em alguns dos estudos, conforme resultados apresentados, que há incidência da doença renovascular aterosclerótica em jovens na terceira e quarta décadas de vida. Em nenhum dos estudos houve predominância no sexo feminino. O predomínio no sexo feminino, ao contrário da etiologia aterosclerótica, ocorre na displasia fibromuscular, que representa 20 a 25% do total dos casos^34^.

Quanto às indicações para a realização de tratamento endovascular, não houve uniformidade nas amostras dos estudos. Houve tanto casos em que observamos indicação em sua maioria por hipertensão renovascular isolada, nos estudos de Rodriguez Lopez et al.^16^, Fiala et al.^17^ e Chuan-jun et al.^20^, como casos de nefropatia isquêmica associada a hipertensão, nos estudos de Ruchin et al.^18^ e Zhao et al.^19^. Observamos que há consenso entre os autores em não indicar procedimento intervencionista para casos de pacientes dialíticos. Apenas no estudo de Fiala et al.^17^ foi indicada angioplastia com stent em um paciente com insuficiência renal dialítica, mas não houve nenhuma mudança clínica pós-procedimento.

A história natural da doença aterosclerótica renal associa-se a uma estenose progressiva arterial, piora da função renal gerando nefropatia isquêmica, atrofia do parênquima e oclusão renal. A taxa de progressão da doença estenótica renal moderada sem tratamento pode atingir de 40 a 70% em dois anos e os índices de oclusão renal podem atingir de 11 a 39%^35^^,^ 36. Além disso 20% dos pacientes com estenose severa de artérias renais podem evoluir para atrofia do parênquima em até 2 anos^37^. Uma conduta terapêutica inadequada pode agravar um quadro clínico inicial, não permitindo intervenções em uma fase mais avançada, a exemplo de pacientes que se tornam dialíticos.

Observou-se uma predominância da doença aterosclerótica unilateral, em conformidade com outros autores que descrevem uma prevalência de 53 a 80%^1^^,^ 29 ^,^ 30. Os graus de estenose utilizados como critérios para indicação de tratamento intervencionista continuam sendo divergentes. Os autores variaram suas amostras em indicações primárias variando de 60 a 70%^16^^,^ 21.

Há consenso atualmente na utilização de stents balão expansíveis nas angioplastias de artérias renais, devido à precisão na liberação e à força radial nas lesões ateroscleróticas. Entre os estudos que descreveram os tipos de stents utilizados, houve um predomínio dos balões expansíveis^16^^,^ 17 ^,^ 20 ^,^ 21. Apenas três estudos fazem referência à marca e ao modelo utilizado. Sabe-se que houve uma evolução tecnológica muito grande até o presente momento comparativamente aos materiais endovasculares utilizados na década de 80 e 90. Os stents apresentaram mudanças em relação às ligas metálicas utilizadas e à sua arquitetura, refletindo diretamente nos resultados em termos de taxas de complicações, perviedade e durabilidade.

Com relação ao número de stents utilizados nos estudos, pudemos observar a necessidade de mais de um stent por artéria em quatro estudos. Isso reflete as características das lesões estenóticas, que podem ser extensas ou apresentar grau de estenose residual ao término do procedimento. Quanto ao número de stents utilizados por paciente, isso pode ser justificado pela presença de estenose bilateral.

Dos desfechos clínicos primários avaliados na literatura, em relação aos procedimentos intervencionistas das artérias renais, há uma predominância da análise da função renal e dos níveis pressóricos^1^^,^ 29 ^,^ 30. A função renal pode ser mensurada através dos níveis de creatinina pré e pós-procedimento, bem como pelo clearance de creatinina estimado pela fórmula de Cockcroft-Gault ou pela urina de 24 horas. Através dessas informações, há uma estratificação clínica dos pacientes, permitindo uma avaliação ao longo do tempo. Dos estudos incluídos na presente revisão sistemática, observamos haver uma queda a longo prazo nos níveis de creatinina nos estudos de Fiala et al.^17^ e Ruchin et al.^18^, com significância estatística. Houve um aumento dos níveis de creatinina, em relação ao pré-operatório, no estudo de Chuan-jun et al.^20^, mas sem significância estatística.

Com relação aos desfechos clínicos e sua frequência, observa-se haver na maioria dos casos uma estabilização da função renal. Em segundo lugar, observa-se uma melhora da função renal. A minoria dos casos apresenta piora da função renal, demonstrando que os resultados da angioplastia de artérias renais a longo prazo são benéficos para o funcionamento renal. Sob o ponto de vista fisiopatológico, a angioplastia de artérias renais gera uma melhora do fluxo renal, com melhora da retenção hídrica e diminuição da sobrecarga renal^38^.

Com relação aos níveis pressóricos, os estudos incluídos demonstraram haver uma melhora dos níveis pressóricos (PAM, PAS e PAD) no pós-procedimento em relação ao pré-procedimento. Isso comprova-se também pela diminuição no número de classes de anti-hipertensivos utilizados no pré e pós-procedimento tardio, demonstrando que os resultados precoces se mantêm ao longo do tempo. O fato de a diminuição do número de classes de anti-hipertensivos ocorrer associada à redução dos níveis pressóricos representa um avanço no controle clínico global dos pacientes. Com a redução da hipertensão arterial há uma diminuição da morbimortalidade.

A taxa de perviedade dos estudos foi, semelhante, variando de 79,2 a 90% entre 24 a 36 meses, exceto no estudo de Fiala et al.^17^ em que a perviedade mostrou-se com valores abaixo dos demais. Porém, podemos observar que nesse estudo clínico a gravidade da doença renal é maior. Pode-se inferir nesse estudo, pela época de sua realização, entre outubro de 1994 e dezembro de 1996, que muitos dos materiais endovasculares estavam sendo desenvolvidos^17^.

Os estudos incluídos nesta revisão apresentam baixos índices de perda de seguimento das amostras, levando em consideração o longo período de seguimento avaliado.

## CONCLUSÃO

A realização desta revisão sistemática mostrou que existem poucas evidências científicas de qualidade na literatura atual, conforme pesquisa nas bases de dados eletrônicas pesquisadas, quanto aos resultados a longo prazo da angioplastia com stent de artérias renais na doença renovascular de etiologia aterosclerótica, além de reduzido número de artigos com metodologias homogêneas. Os estudos não permitem responder aos desfechos propostos de maneira clara e objetiva. Porém, permitem discutirmos o que há na literatura atual e observarmos os resultados que se repetem nos estudos, podendo assim sugerir tendências nas condutas terapêuticas e seus desfechos. Tais resultados poderão ser confirmados, futuramente, através de estudos clínicos com grupos homogêneos, prospectivos, randomizados, controlados, multicêntricos, com delineamento e metodologias adequadas.

Dos sete artigos incluídos na presente revisão sistemática, apesar das deficiências metodológicas, observou-se que os autores divergem quanto ao grau de estenose utilizado para indicação primária do tratamento endovascular. Além disso, sugere-se haver a longo prazo, com significância estatística nos estudos primários utilizados na presente revisão sistemática, manutenção da estabilidade da função renal, melhora do controle pressórico arterial e diminuição do número de classes de medicamentos anti-hipertensivos.
