# Supplementary material for: Resultados a longo prazo da angioplastia de artérias renais com stent na doença aterosclerótica: revisão sistemática
Source: J Vasc Bras. 2017 Apr-Jun;16(2):150–61. [Article in Portuguese] doi: 10.1590/1677-5449.010816 (PMC5915863; doi:10.1590/1677-5449.010816)
Supplement: Translation

# Translation: Long term outcome of renal artery angioplasty with stenting for atherosclerotic stenosis: a systematic review

**Authors:** Daniel Emilio Dalledone Siqueira, Ana Terezinha Guillaumon

**Affiliations:** 1 Universidade Estadual de Campinas – UNICAMP, Cirurgia Vascular, Campinas, SP, Brazil.

**Keywords:** angioplasty, atherosclerosis, renovascular hypertension, nephropathy, renal artery occlusion

## Abstract

Atherosclerotic renovascular disease is the most important cause of secondary hypertension. The natural history of the disease reveals progression rates of 4 to 12% per year. Angioplasty with renal artery stenting is one treatment option; but there are few studies that have reported long-term results. The objective of this systematic literature review is to discuss the long-term results (at least 24 months) of angioplasty with stenting of renal arteries for atherosclerotic disease, in terms of renal function and blood pressure levels for control of hypertension. A thorough search was conducted of LILACS, EMBASE, SCIELO, Cochrane Library, and MEDLINE using the appropriate terms. Just seven out of 2170 references identified met all inclusion criteria. It was concluded that over the long term renal function was stabilized, blood pressure levels were reduced, and the number of classes of antihypertensive medication decreased.

## INTRODUCTION

Renal-artery stenosis (RAS) can be defined as a multifactorial disease, with a number of different etiologies, that can involve the renal arterial vasculature unilaterally or bilaterally and causes varying degrees of stenosis, from the origin to the hilum. Clinically, it can present as renovascular hypertension and ischemic renal disease, causing long-term systemic complications.^1^^,^ 2 This pathology is associated with increased cardiovascular risk and elevated mortality.^3^^-^ 6 For the purposes of research and treatment, many authors define renal artery stenosis as critical when it is greater than 60%.^7^ However, there is considerable variation in the literature and some authors consider stenosis greater than 50% to be critical.^1^ Diagnostic and laboratory methods, in combination with technological advances in noninvasive examination methods, have led to an increase in the number of cases diagnosed, enabling therapeutic management to be initiated earlier.

There as several controversial issues related to treatment of atherosclerotic renal disease, on which there is no consensus in the literature.^8^^-^ 10 There are studies that contradict each other on the subject of clinical treatment vs. angioplasty, the majority of which have reported on short-term follow-up. There are few studies in the literature that present and discuss the later clinical results (beyond 24 months) of renal artery angioplasty with stenting.

The prevalence of RAS is controversial, because there is a lack of population studies designed to identify rates and correlate them with race, age, and sex. However, studies have shown that in some populations RAS is present in 1 to 5% of people with systemic arterial hypertension^1^ and is the principal cause of secondary hypertension. Additionally, it is estimated that RAS is responsible for 1% of mild to moderate hypertension cases and 10 to 40% of cases of acute, severe, or refractory hypertension.^11^ Population studies suggest that the prevalence in people over the age of 65 exceeds 7%.^1^

Renal-artery stenosis can have a variety of different etiologies, but the two major causes are atherosclerosis and fibromuscular dysplasia. Atherosclerotic causes are the most common, accounting for 70 to 80% of cases, with greatest incidence in men over the age of 40, and causing stenosis of proximal segments of the renal arteries.^12^

The natural history of atherosclerotic renal disease has not been completely explained, but it is known that there is progressive stenosis, reducing arterial flow and causing loss of renal function.^13^^,^ 14 This loss is directly dependent on the degree of renal artery stenosis. It is estimated that renovascular atherosclerotic disease causes kidney failure in 5 to 15% of adults who become dependent on dialysis each year,^15^ and just 56% of patients who need dialysis survive for more than 2 years.^15^

In view of all of the above and bearing in mind that renovascular atherosclerotic disease is the principal cause of secondary systemic arterial hypertension and that surgical treatments are linked with a series of controversies, it is clear that there is a lack of clinical studies with explanatory outcomes. Furthermore, the studies that do exist have reported divergent and conflicting results, with the result that conclusive data are lacking.

In this context, a systematic review is needed to determine the long term outcomes of renal artery angioplasty for atherosclerotic renovascular disease. A systematic review is a synthesis of information on a specific subject from a wide range of studies. They are conducted using explicit, predetermined, and reproducible methodology. They are very different from narrative reviews, also known as classical reviews, because they conform to the rules of conventional scientific experiments.

The objective of this study was to evaluate and carefully summarize information identified by a systematic review of the literature relating to the long term (beyond 24 months) clinical outcomes, in terms of renal function and blood pressure levels, of renal artery angioplasty with stenting for treatment of renovascular disease of atherosclerotic origin.

## METHODS

A systematic review of the literature to collate and analyze data. The methods recommended by the Cochrane Collaboration were employed.

### Ethical considerations

No confidential or personal data were employed and no research was conducted involving human beings. The entire study was exclusively based on primary clinical studies indexed on electronic databases. It is worth reiterating the fact that the Brazilian National Research Ethics Commission (CONEP - Comissão National de Ética em Pesquisa) has issued a position statements relating to systematic reviews making it clear that studies that only use data that are already in the public domain, that do not identify research participants, or that are exclusively bibliographic reviews and do not involve human beings are exempt from the need to obtain approval via the CEP-CONEP system.

### Time and place of research

This study was designed and conducted at Disciplina de Moléstias Vasculares, Hospital de Clínicas, UNICAMP. The search for references was performed from April to August, 2016.

Inclusion criteria for articles:

- Types of studies included:

Prospective and retrospective clinical studies that evaluated the results of endovascular treatment of renal artery stenosis of atherosclerotic origin conducted by primary angioplasty with stenting were included if they reported mean follow-up of at least 24 months.

- Types of studies excluded:

Clinical studies were excluded if they did not meet the inclusion criteria, such as studies with mean follow-up of less than 24 months; studies dealing with renal stenosis etiologies other than atherosclerosis; studies of treatment by balloon angioplasty, angioplasty without stent placement, angioplasty using mechanical mechanisms for renal protection (embolization filters); studies describing restenosis cases with assisted-primary patency or secondary patency; clinical studies comparing angioplasty with stent implantation. Case reports and studies of drug treatments in which the data for the angioplasty with stenting group were not described separately or clearly were also excluded.

### Patients

Patients with renal artery stenosis of atherosclerotic origin treated with endovascular stent placement.

### Types of intervention

Only studies of endovascular interventions for treatment of renal artery stenosis were included. Clinical studies of any other type of intervention were excluded.

### Clinical outcomes assessed in studies

The following clinical outcomes were assessed: renal function, blood pressure levels, number of classes of antihypertensive drugs, restenosis, number of dialysis patients.

Search strategies used to locate articles:

- Electronic databases:

Searches were run to identify relevant studies on the leading computerized health sciences databases, including: Literatura Latina Americana e do Caribe em Ciências da Saúde (LILACS), Excerpta Medica Database (EMBASE), Cochrane Library, Scientific Electronic Library Online (SciELO), and MEDLINE, via PubMed.

Electronic searches:

Searches were run on the electronic databases using the following terms in English: renal artery, stenosis, angioplasty, atherosclerotic, stent angioplasty, stent balloon, atherosclerotic, long term.

Selection of studies:

In preparation for this article, the two authors responsible for the literature review (DEDS and ATG) independently analyzed the titles and abstracts identified by the searches. Copies of the full text were obtained for all clinical studies that were relevant and/or potentially relevant and evaluated against the criteria for inclusion of articles.

After copies of the full text for all potentially or definitively relevant articles had been obtained, both authors analyzed them and classified each study as follows: studies excluded, studies to be analyzed, or studies included.

There was no blinding to the authors of the articles, institutions, or results of trials during evaluation.

Policy for dealing with missing data:

If data needed for the review were missing in the texts of selected studies, attempts were made to contact the lead investigator of clinical studies. This policy was applied when statistics or data were missing in studies that had been selected. However, it proved impossible to obtain additional information. In view of this, all data were extracted from the data provided in the published documents.

Searches for ongoing studies:

The Cochrane library did not have any systematic reviews in progress on the same topic as this review.

Searches for unpublished studies:

No searches for unpublished studies were undertaken, since this is a systematic review of clinical studies and published data.

Critical evaluation of the studies:

Critical evaluation of the quality of the studies was based on the following criteria: clear definition of the initial objectives and the clinical outcomes measured, assessment of the methodological quality of the study, use of appropriate statistical methods, description of whether the size of the sample to be studied was calculated, description of whether the study was single-center or multicenter, description of sources of financing.

Extraction of data:

After selection of the articles eligible for the study, the texts were read carefully and with attention, with the objective of extracting the relevant data. The extraction process took into consideration the characteristics of the studies and a compilation of the results of each article, using a protocol for data collection.

Spreadsheets were prepared and subjected to pretest with five studies from the same area, but which were not part of this review. During the pretest, no ambiguities or failures were detected and so the spreadsheets were approved for the primary study.

The name of the first author of the article was used to identify each study and the data needed for the calculations for the statistical tests chosen and any other data judged relevant were extracted.

All of the data employed for the review were taken directly from the published articles or calculated from the information provided.

Analysis and interpretation of the data:

To deal with the peculiarities of the characteristics of the clinical studies in terms of sample sizes, heterogeneous data, and missing clinical data, graphs were plotted and tables constructed in order to allow comparison of the clinical data being analyzed, always chosen with the intention of describing the information under analysis in a clear and effective manner.

The data extracted from the clinical studies selected for this systematic review are presented in the form of absolute values and frequencies. This is not a study in which inferences are made, since the data does not come from a unified sample, and the results shown therefore represent all of the material available in the literature that is compatible with the methodology adopted.

## RESULTS

The initial literature searches identified 2,170 references, 324 of which were duplicate records, making an effective total of 1,684 unique references in the electronic databases. After analysis of titles, 1,337 articles were excluded, leaving 347 clinical studies potentially of interest. The abstracts of these 347 articles were read and 88 studies were considered potentially compatible with the study selection criteria. After reading the full texts and conducting an initial assessment, a further 62 articles were excluded and 26 articles selected for further analysis.

All of these 26 articles were subjected to a rigorous evaluation according to the protocol for inclusion and analyzed carefully against detailed criteria. Nineteen of these 26 articles were excluded during this step and seven studies that fully met all of the inclusion criteria for the study remained.

The study search and analysis strategy is illustrated in Figure 1, showing the numbers of articles identified, selected, excluded, and included at each stage.

**Figure 1:** Summary of results of searches on electronic databases.

Methodological quality of the studies included:

All of the studies selected for and included in this systematic review after analysis against the inclusion criteria are retrospective and all have a single intervention group. Therefore, none of them involve blinding of examiners, calculation of sample size, management of follow-up losses, criteria for group allocation, or analysis according to the study authors’ intention to treat.

Analysis of the data:

- Characteristics of the study samples:

After analysis of the studies, according to the methodology described earlier, seven clinical studies^16^^-^ 22 with a total of 705 patients were selected. The samples comprised 375 male patients and 249 female patients, but one study, by Zhao et al.,^19^ did not provide data on the sex of the 81 patients studied (Table 1). The mean age of patients was 68.8 years, ranging from 37 to 87 (Table 2).

**Table 1:** Characteristics of the samples in the clinical studies included in the systematic review, showing sample size and sex distribution for each study.

**Article**	**n**	**Male**		**Female**	
		**n**	**%**	**n**	**%**
Rodriguez Lopez et al.^16^	108	64	59.3	44	40.7
Fiala et al.^17^	21	11	52.4	10	47.6
Ruchin et al.^18^	89	53	59.6	36	40.4
Zhao et al.^19^	81	NA		NA	
Chuan-jun et al.^20^	125	74	59.2	51	40.8
Zeller et al.^21^	241	153	63.5	88	36.5
Bucek et al.^22^	40	20	50.0	20	50.0

NA: Data not available in the primary study.

**Table 2:** Characteristics of the samples in the clinical studies included in the systematic review, showing sample size and distribution by mean age, minimum age, and maximum age, in years, for each study.

**Article**	**n**	**Mean age**	**Minimum age**	**Maximum age**
Rodriguez Lopez et al.^16^	108	72.0	37.0	87.0
Fiala et al.^17^	21	63.0	46.0	87.0
Ruchin et al.^18^	89	70.0	37.0	86.0
Zhao et al.^19^	81	76.2	NA	NA
Chuan-jun et al.^20^	125	66.4	NA	NA
Zeller et al.^21^	241	67.0	44.0	84.0
Bucek et al.^22^	40	65.2	NA	NA

NA: Data not available in the primary study.

Characteristics of the studies

The countries in which the studies were conducted were as follows: two articles are from the United States of America, two from China, one from Australia, one from Germany, and one from Austria. The journals in which the articles were published are shown in Chart 1.

**Chart 1:** Names of authors, years of publication, countries of origin, multi/single center, and name of journal.

**Lead author**	**Year**	**Country of origin**	**Centers**	**Name of Journal**	**Follow-up (months)**
Rodriguez Lopez et al.^16^	1999	United States	Single center	Journal of Vascular Surgery	36
Fiala et al.^17^	1998	United States	Multicenter	Annals of Vascular Surgery	24
Ruchin et al.^18^	2007	Australia	Single center	Heart, Lung and Circulation	28
Zhao et al.^19^	2012	China	Single center	Clinical Interventions in Aging	31.3
Chuan-jun et al.^20^	2012	China	Single center	Chinese Medical Journal	24
Zeller et al.^21^	2003	Germany	Single center	Journal of Endovascular Therapy	27
Bucek et al.^22^	2003	Austria	Single center	Wiener Klinische Wochenschrift	39.6

All seven of the clinical studies included in the systematic review are retrospective, six studies were undertaken at a single center and one is a multicenter study. Chart 1 lists the whether each study was single-center or multicenter, by lead author and year of publication.^16^^-^ 22 Mean follow-up time of the studies was 29.9 months, ranging from 24 to 39.6 months (Chart 1). The sample selection, angioplasty with stenting procedures, and clinical follow-up described in these studies occurred from 1993 to 2010, although the studies were published from 1998 to 2012.

Arteries treated/Types of stents/bilateral vs. unilateral:

In the 705 patients described, 876 arteries were treated with placement of 901 renal stents, equating to mean numbers of stents per patient ranging from 1.0 to 1.57, and mean number of stents per artery of 1.0 to 1.32, depending on the study (Table 3). With relation to the stents employed in the clinical studies, only the studies by Rodriguez Lopez et al.,^16^ Fiala et al.,^17^ and Zeller et al.^21^ mention the brand and model of stent implanted. The other articles only state that balloon expandable stents were used in all procedures.

**Table 3:** Total number of patients per clinical study, number of renal arteries treated, number of stents per study, and mean number of stents per artery and per patient in each clinical study.

**Article**	**n**	**Number of arteries treated**	**Number of stents implanted**	**Number of stents per artery**	**Number of stents per patient**
Rodriguez Lopez et al.^16^	108	125	125	1.15	1.15
Fiala et al.^17^	21	25	33	1.32	1.57
Ruchin et al.^18^	89	102	110	1.08	1.25
Zhao et al.^19^	81	86	86	1.00	1.06
Chuan-jun et al.^20^	125	143	143	1.00	1.14
Zeller et al.^21^	241	355	364	1.02	1.51
Bucek et al.^22^	40	40	40	1.00	1.00

In the samples from the studies included in this review, renal artery stenosis was predominantly unilateral, accounting for 76.5% of the total patient sample. The articles did not mention cases of single kidney. Additionally, the studies did not agree on the minimum degree of stenosis considered an indication for endovascular treatment with implantation of a stent, varying from 60 to 70%.

Indications for procedures:

The clinical indications for performing the endovascular procedure were distributed between hypertension without ischemic nephropathy, hypertension with ischemic nephropathy, and renal failure requiring dialysis (Table 4). In all studies, just one patient had renal failure requiring dialysis, who was part of the sample studied by Fiala et al.^17^ This patient did not exhibit any type of improvement after the procedure, whether in terms of renal function or control of blood pressure. With relation to the endovascular procedures performed, only Rodriguez Lopez et al.^16^ and Zeller et al.^21^ discussed the arterial access routes used for the procedure, with a predominance of femoral access, followed by brachial access.

**Table 4:** Clinical indications for the endovascular procedure.

**Article**	**n**	**Hypertension**		**Renovascular hypertension in isolation (without ischemic nephropathy)**		**Ischemic nephropathy (renal failure not requiring dialysis) + hypertension**		**Renal failure requiring dialysis**	
		**n**	**%**	**n**	**%**	**n**	**%**	**n**	**%**
Rodriguez Lopez et al.^16^	108	96	88.9	64	59.3	32	29.6	0	0.0
Fiala et al.^17^	21	18	85.7	11	52.4	8	38.1	1	4.8
Ruchin et al.^18^	89	77	86.5	21	23.6	56	62.9	0	0.0
Zhao et al.^19^	81	81	100.0	10	12.3	71	87.7	0	0.0
Chuan-jun et al.^20^	125	118	94.4	79	63.2	39	31.2	0	0.0
Zeller et al.^21^	241	239	99.2	NA	NA	33	13.7	0	0.0
Bucek et al.^22^	40	40	100.0	NA	NA	10	25.0	0	0.0

NA: Data not available in the primary study.

- Renal function

With regard to renal function after the procedures, some of the studies described clinical outcomes that could be stratified in relation to baseline renal function in terms of worse, stabilized, or improved renal function, and all of these data were for the end of the studies’ clinical follow-up periods (Table 5). Creatinine levels before the procedure, after the procedure, within 30 days, and long term after the procedure are given in mg/dL (Table 6).

**Table 5:** Clinical outcomes of each study - renal function.

**Article**	**n**	**Worsened**		**Stabilized**		**Improved**	
		**n**	**%**	**n**	**%**	**n**	**%**
Rodriguez Lopez et al.^16^	108	5	4.6	103	95.4	0	0.0
Fiala et al.^17^	21	1	4.8	13	61.9	7	33.3
Ruchin et al.^18^	89	NA	NA	NA	NA	NA	NA
Zhao et al.^19^	81	13	16.0	26	32.1	8	9.9
Chuan-jun et al.^20^	125	23	18.4	56	44.8	31	24.8
Zeller et al.^21^	241	NA	NA	NA	NA	NA	NA
Bucek et al.^22^	40	10	25.0	NA	NA	NA	NA

NA: Data not available in the primary study.

**Table 6:** Creatinine levels in mg/dL before and after procedure (within 30 days and late follow-up).

**Article**	**Preoperative**		**Postoperative (< 30 days)**		**p**	**Late follow-up**		**p**
	**n**	**%**	**n**	**%**		**n**	**%**	
Rodriguez Lopez et al.^16^	2		1.8		NA	NA		NA
Fiala et al.^17^	1.47	0.57	NA		NA	1.31	0.41	0.076
Ruchin et al.^18^	1.58	0.07	NA		NA	1.47	0.68	0.16
Zhao et al.^19^	1.46	0.63	NA		NA	NA		NA
Chuan-jun et al.^20^	1.66	1.04	1.71	1.08	>0.05	1.77	1.15	> 0.05
Zeller et al.^21^	NA		NA		NA	NA		NA
Bucek et al.^22^	NA		NA		NA	1.3	0.4	NA

NA: Data not available in the primary study.

- Blood pressure levels

With relation to systemic arterial blood pressure, studies assessed the number of antihypertensive drug classes being taken by patients before the procedures and in the late postoperative period (Table 7), mean arterial blood pressure (MBP), systolic arterial blood pressure (SBP), and diastolic arterial blood pressure (DBP) (Tables 8, 9, and 10).

**Table 7:** Number of classes of antihypertensive drugs in preoperative and late postoperative periods.

**Article**	**Preoperative**		**Postoperative (late)**		**p**
	**n**	**%**	**n**	**%**	
Rodriguez Lopez et al.^16^	NA		NA		NA
Fiala et al.^17^	3.1	0.13	2.7	0.25	0.056
Ruchin et al.^18^	3.14	1.65	2.62	1.39	0.05
Zhao et al.^19^	2.28	1.18	2.1	1.0	< 0.01
Chuan-jun et al.^20^	2.7	1.1	1.6	1.1	< 0.05
Zeller et al.^21^	NA		NA		NA
Bucek et al.^22^	NA		NA		NA

NA: Data not available in the primary study.

**Table 8:** Mean arterial blood pressure in mmHg.

**Article**	**Preoperative**		**Postoperative (< 30 days)**		**p**	**Postoperative (late)**		**p**
	**n**	**%**	**n**	**%**		**n**	**%**	
Rodriguez Lopez et al.^16^	NA		NA		NA	NA		NA
Fiala et al.^17^	117	13.4	NA		NA	113	12.8	0.002
Ruchin et al.^18^	NA		NA		NA	NA		NA
Zhao et al.^19^	NA		NA		NA	NA		NA
Chuan-jun et al.^20^	NA		NA		NA	NA		NA
Zeller et al.^21^	NA		NA		NA	NA		NA
Bucek et al.^22^	NA		NA		NA	NA		NA

NA: Data not available in the primary study.

**Table 9:** Systolic arterial blood pressure in mmHg.

**Article**	**Preoperative**		**Postoperative (< 30 days)**		**p**	**Postoperative (late)**		**p**
	**n**	**%**	**n**	**%**		**n**	**%**	
Rodriguez Lopez et al.^16^	NA		NA		NA	NA		NA
Fiala et al.^17^	NA		NA		NA	NA		NA
Ruchin et al.^18^	161.7	29.5	138.2	20.5	< 0.0001	138.7	7.9	< 0.0001
Zhao et al.^19^	155.9	22.8	130.3	4.5	< 0.01	135	4.7	< 0.01
Chuan-jun et al.^20^	168	23	138	17	< 0.05	141	20	< 0.05
Zeller et al.^21^	NA		NA		NA	NA		NA
Bucek et al.^22^	NA		NA		NA	NA		NA

NA: Data not available in primary study.

**Table 10:** Diastolic arterial blood pressure in mmHg.

**Article**	**Preoperative**		**Postoperative (< 30 days)**		**p**	**Postoperative (late)**		**p**
	**n**	**%**	**n**	**%**		**n**	**%**	
Rodriguez Lopez et al.^16^	NA		NA		NA	NA		NA
Fiala et al.^17^	NA		NA		NA	NA		NA
Ruchin et al.^18^	78.4	13.8	69.0	11.6	< 0.003	76.7	10.8	0.62
Zhao et al.^19^	79.3	10.8	66.7	8.9	< 0.01	68.3	10.2	< 0.01
Chuan-jun et al.^20^	92	12	78	10	< 0.05	80	11	< 0.05
Zeller et al.^21^	NA		NA		NA	NA		NA
Bucek et al.^22^	NA		NA		NA	NA		NA

NA: Data not available in the primary study.

- Follow-up losses

Table 11 lists losses from the study samples over the follow-up periods.

**Table 11:** Initial number of patients, number of patients at follow-up and rates of loss.

**Article**	**n**	**Follow-up (months)**	**Initial number of patients**	**Number of patients at follow-up**	**Loss (%)**
Rodriguez Lopez et al.^16^	108	36	108	89	17.6
Fiala et al.^17^	21	24	21	16	23.8
Ruchin et al.^18^	89	28	89	81	9.0
Zhao et al.^19^	81	31.3	81	47	42.0
Chuan-jun et al.^20^	125	24	125	110	12.0
Zeller et al.^21^	241	27	241	198	17.8
Bucek et al.^22^	40	39.6	40	40	0.0

## DISCUSSION

This systematic literature review provides the most up-to-date scientific evidence available on the long-term results of angioplasty with stenting for atherosclerotic disease of the renal arteries, from studies with a minimum follow-up of 24 months. To date, there had not been any systematic reviews or meta-analyses compiling the long-term results of angioplasty.

Certain considerations should be made on the methodological quality of the studies included in this review. The studies available in the literature used a range of different methodologies and the clinical outcomes assessed are not uniform. All of the primary studies that met the inclusion criteria are retrospective. The follow-up times of prospective clinical trials were short, i.e. less than 24 months.^1^^,^ 12

Richard Bright described the combination of arterial hypertension and parenchymatous kidney disease at Guy’s Hospital, in London, in 1836. He observed in autopsies that patients with renal parenchyma abnormalities had enlarged cardiac chambers.^23^ This observation was the initial stimulus for Traube’s speculative description, in 1871, of a mechanical process in which, in the presence of high blood pressure, the heart would be obliged to exert increased contractile force to make blood flow through the distal parts of the vascular system, leading to myocardial hypertrophy.^24^ Bright’s initial description prompted several authors to attempt to recreate the clinical findings in experimental models. In 1934, Harry Goldblatt demonstrated that the initial event was constriction of the renal arteries, triggering arterial hypertension, renal atrophy, and cardiac hypertrophy.^25^

The first treatment described for renovascular hypertension was nephrectomy, since there were no techniques for reconstruction of arteries. This was a definitive method and was associated with high morbidity and mortality. As techniques improved, methods were developed during the twentieth century for renal revascularization using endarterectomy and, later, vascular grafting. These remained current and predominant up to the end of the 1970s. When Grüntzig described his invention for balloon angioplasty in 1978, endovascular treatment became possible and a revolution in treatment methods began.^26^

During the 1980s, clinical studies predominated with the objective of comparing the results of two treatment methods, open surgery vs. balloon angioplasty, for a wide range of etiologies stenotic renal artery disease. Many studies demonstrated the superiority of endovascular procedures, with lower rates of morbidity.^1^

As the years passed, in the 1990s it became clear that the results of balloon angioplasty were unsatisfactory in atherosclerotic disease, with high restenosis rates and the need for endovascular reinterventions.^27^ During this period, studies began to be published investigating a new method for treatment, stent angioplasty, which became the first choice option for atherosclerotic disease, with results that were superior to both open surgery and balloon angioplasty.^28^

A little more than a decade ago, advances achieved by the pharmaceutical industry, developing new molecules and drugs, began a worldwide move to compare the benefits of clinical treatment to interventional treatment. This resulted in prospective clinical trials being run in many countries. However, many of the studies have serious methodological failures, calling into question the results they report.

One of these large-scale clinical studies was the *Stent placement in patients with atherosclerotic renal artery stenosis and impaired renal function: a randomized trial* (STAR study), in which stents were only implanted in part of the intervention group and angioplasty was reserved as a salvage measure for a group on clinical treatment in cases of refractory hypertension, malignant hypertension, or acute pulmonary edema.^29^ In the Angioplasty and Stenting for Renal Artery Lesions (ASTRAL) study, there was variation in the degree of stenosis used to indicate the procedure and patients were excluded at study outset if there was a need for revascularization.^30^ The Cardiovascular Outcomes in Renal Atherosclerotic Lesions (CORAL) study optimized clinical measures excessively, used a filter for protection against microembolization, and excluded patients who had been hospitalized for heart failure during the 30 days leading up to enrollment on the study.^1^ It is known that acute pulmonary edema and heart failure are aggravated by renal artery stenosis.^1^^,^ 11

To date, there are no guidelines setting out the indications for clinical or surgical treatment of renovascular disease.^8^^,^ 10 There are only guidelines from the American Heart Association and the American College of Cardiology giving indications for population screening.

However, the greatest problem with all of the studies published to date is that they do not discuss late results, since mean follow-up periods are short and cannot therefore reflect long-term results. Once stent placement is indicated, one of the most important questions is the long-term outcome.

Several services have based their routines exclusively on the results of clinical trials such as STAR,^29^ ASTRAL,^30^ and CORAL,^1^ without taking a more critical view on these studies, which could lead to conflicting management and results over the long term, since when atherosclerotic renal artery disease is bilateral, it is associated with a mortality rate of 52% in 4 years.^31^^,^ 32 This demonstrates the need to take some type of clinical measure immediately after diagnosis, with the objective of modifying the natural history of the disease.

Among the clinical studies included in the present systematic review, only the study by Fiala et al.^17^ was multi-center, and all were retrospective. The evidence level of retrospective studies is much lower than for prospective studies, but they do offer a basis for discussion of the methods employed, the procedures initiated, and the results observed.

Among the patients treated in the studies selected for this systematic review, there was a predominance of male patients in their sixth decade of life.^33^ This coincides with the peak incidence of atherosclerotic disease, in common with other arterial territories, such as the coronary and carotid regions. However, in some of the studies, as shown by the results reported, it was observed that there is an incidence of atherosclerotic renovascular disease in younger people, still in their third and fourth decades of life. None of the studies had a majority of female patients. In contrast with atherosclerotic etiology, females predominate among cases caused by fibromuscular dysplasia, which is responsible for 20 to 25% of all cases.^34^

There was no uniformity across the different studies’ samples in terms of their indications for endovascular treatment. In some, the majority of cases were treated for renovascular hypertension in isolation, as in the studies by Rodriguez Lopez et al.,^16^ Fiala et al.,^17^ and Chuan-jun et al.,^20^ while in others, the indication was ischemic nephropathy associated with hypertension, as in the studies by Ruchin et al.^18^ and Zhao et al.^19^ We observed that there was consensus between different authors on not indicating interventional procedures for dialysis patients. The only exception was in the study by Fiala et al.,^17^ in which angioplasty with stenting was performed for one patient with renal failure requiring dialysis, but there was no clinical change after the procedure.

The natural history of atherosclerotic renal disease is associated with progressive arterial stenosis, deterioration of renal function with ischemic nephropathy, atrophy of the parenchyma, and renal occlusion. The progression rate of untreated moderate stenotic renal disease can reach 40 to 70% over 2 years and rates of renal occlusion can reach 11 to 39%.^35^^,^ 36 Additionally, 20% of patients with severe stenosis of renal arteries can progress to parenchymal atrophy within 2 years.^37^ Inadequate therapeutic management can exacerbate initial clinical status, ruling out interventions at more advanced phases, as in the case of patients who require dialysis.

It was observed that unilateral atherosclerotic disease predominated, in common with other authors, who describe prevalence rates of 53 to 80%.^1^^,^ 29 ^,^ 30 The degree of stenosis used as criterion for indication of interventional treatment remains a point of divergence. The authors of these studies selected samples with primary indications varying from 60 to 70%.^16^^,^ 21

There is currently consensus on employment of expandable balloon stents for angioplasty of renal arteries, because they offer precise release and radial strength in atherosclerotic lesions. Among studies that described the types of stents employed, there was a predominance of expandable balloon stents.^16^^,^ 17 ^,^ 20 ^,^ 21 Only three of the studies stated the brand and model employed. There has been considerable technological progress in comparison with the endovascular materials used in the 1980s and 1990s. Stents have undergone changes in the metallic alloys employed and in their architecture, reflecting directly on the results in terms of rates of complications and patency and of durability.

With relation to the number of stents used in the studies, it was observed that there was a need to use more than one stent per artery in four studies. This is a reflection of the characteristics of the stenotic lesions, which may be extensive or may still exhibit residual stenosis at the end of the procedure. The number of stents used per patient may be because of bilateral stenosis.

The primary clinical outcomes evaluated in the literature on interventional procedures in renal arteries are predominantly analyses of renal function and blood pressure levels.^1^^,^ 29 ^,^ 30 Renal function can be measured in terms of creatinine levels before and after the procedure, or by creatinine clearance estimated using the Cockcroft-Gault formula or by 24-hour urine. This information can be used to conduct clinical stratification of the patients, enabling long-term assessments. Among the studies included in this systematic review, we observed that there was a statistically significant long-term reduction in creatinine levels in the studies by Fiala et al.^17^ and Ruchin et al.^18^ There was an increase in creatinine levels compared to the preoperative baseline in the study but Chuan-jun et al.,^20^ but the difference was not statistically significant.

With relation to clinical outcomes and their frequencies, it was observed that in the majority of cases renal function was stabilized. The second most common outcome was improved renal function and in a minority of cases renal function worsened, demonstrating that the long-term results of renal artery angioplasty are beneficial to renal function. From the pathophysiologic point of view, angioplasty of the renal arteries improves renal flow, with improvement of water retention and a reduction in renal overload.^38^

With relation to blood pressure, the studies included reported improvements in blood pressure levels (MBP, SBP and DBP) after the procedure in relation to before the procedure. This was also shown by a reduction in the number of classes of antihypertensive drugs from before the procedure to late postoperative follow-up, demonstrating that early results were maintained over the longer term. The fact that the number of antihypertensive classes was reduced in combination with the lower blood pressure levels represents an improvement in global clinical control for these patients. With reduced arterial hypertension comes lower morbidity and mortality.

Patency rates were similar in these studies, varying from 79.2 to 90% at 24 to 36 months, with the exception of the study by Fiala et al.,^17^ in which patency rates were lower than in the other studies. However, the severity of kidney disease was greater in that clinical study and it can be inferred from the dates of the study, conducted from October 1994 to December 1996, that many of the endovascular materials were still being developed.^17^

The studies included in this review have low follow-up losses from their samples, considering the length of follow-up period involved.

## CONCLUSIONS

This systematic review has shown that there is little high-quality scientific evidence in the current literature (according to the electronic databases searched) on the long-term results of angioplasty with stenting of renal arteries for renovascular disease of atherosclerotic etiology, and the number of articles with uniform methodologies is small. The studies that do exist did not enable the review’s objectives to be achieved with clear and objective answers for the outcomes selected. However, they do provide a basis for discussing what is available in the current literature and for observing which results are repeated in the studies, indicating trends in therapeutic management and its outcomes. These results could be confirmed in the future by prospective, randomized, controlled, multicenter clinical studies with homogenous groups and appropriate methodologies.

The seven articles included in this systematic review exhibited certain methodological deficiencies, but it was nevertheless possible to observe that their authors diverge in terms of the degree of stenosis used as a primary indication for endovascular treatment. Notwithstanding, the primary studies analyzed in the present systematic review suggest that, over the long term and with statistical significance, there was maintenance of the stability of renal function, improvement of blood pressure control, and reduction in the number of classes of antihypertensive medications.
